# Special Issue: Sudden Cardiac Death: Clinical Updates and Perspectives

**DOI:** 10.3390/jcm11113120

**Published:** 2022-05-31

**Authors:** Tobias Schupp, Ibrahim Akin, Michael Behnes

**Affiliations:** First Department of Medicine, University Medical Centre Mannheim (UMM), Faculty of Medicine Mannheim, University of Heidelberg, 68167 Mannheim, Germany; tobias.schupp@umm.de (T.S.); ibrahim.akin@umm.de (I.A.)

Sudden cardiac death (SCD) is defined as unexpected sudden death due to cardiac causes, occurring within one hour after the onset of symptoms [1]. In up to 50%, SCD occurs as initial manifestation of coronary artery disease (CAD) or other structural heart disease. The incidence of SCD has significantly decreased by 17% in men and 31% in women from 1997 to 2010 [2]. This may be related to the prognostic benefit of an implantable cardioverter-defibrillator (ICD), as well as important pharmacotherapies for the prevention of ventricular tachyarrhythmias (including beta-blockers, angiotensin converting enzyme inhibitors (ACEi), receptor blockers (ARB) and mineralocorticoid receptor antagonists (MRA)). Their prognostic impact has already been demonstrated within large randomized-controlled trials (RCT), leading to their implementation within current European guidelines with a class I indication and a level of evidence A [1,3]. By now, more than 38,000 articles on the topic “sudden cardiac death” are available on PubMed central. Due to the overall decreasing rates of SCD, the high evidence of guideline-recommended therapies and the overall increasing number of articles on the topic of SCD, one may therefore question whether updates on this “*old topic*” are necessary and worth a Special Issue?

Taking an in-depth view on the indication of pharmacological therapies reducing overall all-cause mortality and specifically SCD rates, it becomes apparent that most of the guideline relevant RCT—such as the “MERIT-HF”, “CIBIS-II” and the “SOLVD” study—were published at the end of the last century [4,5,6]. For instance, the “CIBIS-II” study demonstrated decreased risk of all-cause mortality and SCD in 2647 heart failure (HF) patients with left ventricular ejection fraction (LVEF) of 35% or less treated with bisoprolol as compared to placebo at 1.3 years [4]. Although 96% of patients received concomitant treatment with an ACEi, the rate of digitalis treatment was 52%, which was shown not to improve cardiovascular mortality, leading to a significant decline of prescription rates over the past decade [7,8]. On the other hand, patients in the “CIBIS-II” study were median-aged 61 years, which may be partly related to exclusion criteria in RCT, but furthermore reflects the ongoing demographic changes and changes of patients’ characteristics with cardiovascular diseases. Despite improvements of nationwide healthcare supply, adherence to international guideline recommendations and better coronary revascularization strategies have led to an older population of patients with cardiovascular diseases with an increased burden of comorbidities (such as atrial fibrillation, chronic kidney disease and severe HF) [9,10]. In line with this, the clinical presentation of SCD has also changed and far more patients present with an initial non-shockable rhythm, which reflects the improvements in diagnosis and treatment of structural heart disease [9]. However, only one RCT, the “PARADIGM-HF” trial, recently investigated the prognostic impact of pharmacotherapies regarding SCD. Thus, treatment with the angiotensin receptor–neprilysin inhibitor LCZ696 reduced the risk of SCD irrespective of the presence of an ICD despite optimal medical treatment [11,12]. Since the prognostic value of established pharmacotherapies remains questionable in the modern medicine era and no RCT are currently on the way to re-evaluating their prognostic impact, European guidelines demand the need for registry data [1]. Therefore, this Special Issue of the *Journal of Clinical Medicine* aims to provide insights on current research, focusing on the identification of patients at risk for SCD, as well as on the prognostic impact of diagnostic and therapeutic tools in patients with cardiac arrest or ventricular tachyarrhythmias, who are at highest risk of SCD. Currently, five studies have been published within the current Special Issue.

One study by Kim et al. investigated the prognostic impact of metabolic syndrome and gamma-glutamyl transferase (ɣ-GTP) on SCD. Including more than 4,000,000 patients undergoing nationwide health screenings in Korea, they demonstrated metabolic syndromes and elevated ɣ-GTP associated with increased SCD risk. It is of note that decreasing ɣ-GTP during follow-up has been shown to reduce the risk of SCD, which may be related to the effect of lifestyle modification [13]. These findings are important since risk stratification for SCD in clinical practice predominantly relies on LVEF. However, it was demonstrated that most SCD cases occur in patients with no evidence of depressed LVEF, who are considered as “*low risk*”, with no evidence of structural heart disease, which makes the identification of risk factors for SCD even more complicated [9]. Thus, especially in HF-related SCD, rates have improved due to ICD implantation and pharmacotherapies, whereas SCD decline was much lower in patients without depressed EF and without prior AMI [14]. Therefore, the study by Kim et al. is a relevant step to develop an improved SCD risk stratification tool within the general population at low risk of SCD.

Even fewer data are available that focus on diagnostic and therapeutic approaches in patients surviving aborted cardiac arrest (i.e., for the secondary prevention of SCD). Although these patients are at the highest risk of suffering from SCD, all RCT investigating prognosis of heart failure therapies included patients without HF or structural heart disease and without prior ventricular tachyarrhythmias (i.e., primary prevention of SCD) [4,5]. It is of note that only RCT demonstrating the prognostic superiority of an ICD included patients for secondary prevention of SCD [15]. Using a large registry of over 2400 patients, we recently identified age, sex, as well as important comorbidities (such as chronic kidney disease, LVEF, AMI, CAD) to predict outcomes following ventricular tachyarrhythmias [16,17,18]. Recently, biomarkers have gained more importance in predicting prognosis in patients with HF and AMI. In this Special Issue, we demonstrated that cardiac troponin I is a useful predictor of short-term mortality within 30 days following ventricular tachyarrhythmias, which was observed in both patients with and without CAD and AMI [19] This underlines the importance of cardiac troponins for the prediction of prognosis in high-risk patients despite their implementation in the diagnosis of AMI. With regard to pharmacotherapies, we were also able to demonstrate comparable benefit of ACEi as compared to ARB treatment following ventricular tachyarrhythmias, which is in line with prior studies including patients with AMI or HF [20,21,22]. ACEi and ARB were investigated in former studies including patients with AMI or HF, whereas again re-evaluation of “established” pharmacotherapies for the prevention of SCD within the current era of modern cardiovascular medicine is demanded in current European guidelines [1].

Within this Special Issue, two studies included patients with out-of-hospital cardiac arrests (OHCA). A study based on the “JAAM Out-Of-Hospital Cardiac Arrest registry” by Nojima et al. suggested blood ammonia levels at hospital arrival were useful to predict neurological outcomes following OHCA, taking into account whether the return of spontaneous circulation (ROSC) was achieved at hospital admission [23]. Given the overall poor prognosis of patients with OHCA, especially in the setting of refractory OHCA, these findings are important for the early identification of patients with presumably favorable outcomes. In line with this, Rysz et al. demonstrated within a propensity-score matched study of 940 OHCA patients from Sweden, that inotropic support with levosimendan was only used in 10% of OHCA patients and was not associated with favorable outcomes; however, a small subgroup of patients treated with levosimendan <6 h had improved mortality. Despite the overall limited data with regard to levosimendan use following cardiac arrest, further studies are warrened to identify patients that may benefit from levosimendan therapy [24]. Besides the prognostic impact of inotropic agents in patients with OHCA or cardiogenic shock, the use of mechanical circulatory support (MCS) devices may improve the in-hospital survival of these patients. Although the “ARREST” trial randomized only 30 patients to extracorporeal membrane oxygenation (ECMO) or standard treatment, improved survival until hospital discharge was shown in patients undergoing ECMO therapy [25]. On the contrary, the randomized controlled ECLS-shock trial (clinicaltrials.gov identifier: NCT03637205) is currently investigating the prognosis of ECMO therapy in patients presenting with cardiogenic shock. Considering the limited evidence from RCT, the investigation of both invasive strategies and pharmacological therapies in OHCA needs further investigation. 

In conclusion, evidence regarding the prediction of SCD and treatment strategies of patients at high risk of SCD are scarce, despite the overall high number of studies in this field. This is related to ongoing demographic changes, improvements of HF and AMI therapies and the overall difficult scenario of developing appropriate SCD risk prediction models, which is related to the high absolute number of SCD occurring in patients with no evidence of structural heart disease or severe HF. However, the present Special Issue may provide further insights into SCD prevention and the treatment of OHCA/ventricular tachyarrhythmias in the current era of medicine.

As Guest Editors of this Special Issue, we would like to thank the authors for their valuable contributions and the *Journal of Clinical Medicine* Editorial Office for their continuous support.
